# Role of mitophagy in acute and fractionated gamma radiation–induced nephropathy in rats: insight into molecular biology and repurposing of rosuvastatin

**DOI:** 10.1007/s00210-025-04901-6

**Published:** 2025-12-29

**Authors:** Noha A. Fadel, Dina M. Lotfy, Asmaa A. Gomaa, Abeer Bishr

**Affiliations:** 1 Drug Radiation Research Department, National Centre for Radiation Research and Technology (NCRRT), Egyptian Atomic Energy Authority (EAEA), Cairo, Egypt; 2 Pharmacology and Toxicology Department, Faculty of Pharmacy, Ahram Canadian University, Giza, Egypt

**Keywords:** Rosuvastatin, Acute irradiation, Fractionated irradiation, Nephropathy, Mitophagy, Apoptosis

## Abstract

Radiotherapy is crucial in curative oncology, but normal tissue injuries, such as the kidney, restrict its usage. While rosuvastatin (ROSU) is experimentally known to mitigate renal damage, its potential role in protecting against radiation-induced nephrotoxicity has never been investigated. Accordingly, the current study explored the ROSU’s protective impact against radiation-induced nephropathy, with a particular focus on mitophagy regulation. Animals were exposed to 8 Gray (Gy) of whole-body gamma irradiation, either acute or fractionated (2 Gy × 4), and received ROSU (10 mg/kg, i.p.) pre- and post-radiation. Kidney injury was assessed by estimating kidney functions, oxidative stress parameters, and histopathological alterations. To elucidate the mechanism of ROSU, the gene and protein expression of sirtuin 1 (SIRT1) and forkhead box class O (FOXO3a) were estimated, alongside mitophagy and apoptotic biomarkers. Radiation exposure induced cellular necrosis and apoptosis, impaired renal function, and oxidative imbalance. ROSU treatment markedly ameliorated these alterations, demonstrating potent antioxidant activity, as evidenced by reduced malondialdehyde (MDA) level and elevated reduced glutathione (GSH), glutathione peroxidase (GPx), and superoxide dismutase (SOD) levels. Mechanistically, ROSU activated SIRT1 and promoted FOXO3a deacetylation, thereby restoring radiation-impaired mitophagy, as indicated by increased expression of PTEN-induced putative kinase protein 1 (PINK1), Parkinson protein 2 E3 ubiquitin protein ligase (Parkin), and autophagy-related gene 5 (ATG5). This was accompanied by the suppression of intrinsic apoptosis triggered by radiation, as shown by decreased cleaved caspase-3 expression. This study repurposes ROSU in modulating radiation-induced nephropathy, revealing its novel role in redirecting cell fate from apoptosis toward mitophagy through SIRT1/FOXO3a activation.

## Introduction

About 60% of cancer patients undergo ionizing radiation as part of their therapeutic approach, making radiotherapy a cornerstone in the treatment of numerous malignancies (Hall et al. 2016). Although radiation is now more precise, off-target effects on adjacent healthy tissues remain a significant limitation (Verginadis et al. 2025). Radiation-induced nephropathy is a significant complication in oncological radiotherapy, occurring in patients treated for lymphomas, gastrointestinal and gynecological cancers, and in those receiving total body irradiation for bone marrow transplantation, potentially leading to progressive and irreversible renal impairment (Dawson et al. 2010; Klaus et al. 2021). The pathophysiology of radiation-induced nephropathy is driven by direct DNA double-strand breaks and reactive oxygen species (ROS)-mediated oxidative stress, which trigger inflammation and apoptosis, ultimately resulting in nephron mass loss and interstitial fibrosis (Ahmad et al. 2023; Klaus et al. 2021).

Among the body’s intrinsic protective repair responses, autophagy is a conserved cellular degradation mechanism crucial for homeostasis, enabling the recycling of damaged organelles, misfolded proteins, and toxic aggregates (Abdrakhmanov et al. 2020). Mitophagy is a specialized form of autophagy that helps to preserve mitochondrial integrity by selectively removing damaged or defective mitochondria. This process inhibits the accumulation of excessive ROS, thereby maintaining cellular homeostasis (Onishi et al. 2021). Two major mitochondrial priming mechanisms have been identified; one is dependent on the PTEN-induced putative kinase protein 1 (PINK1)/Parkinson protein 2 E3 ubiquitin protein ligase (Parkin). The other is the mitophagy receptor-mediated route, which depends on the autophagy receptor proteins found on mitochondria (Ashrafi and Schwarz 2013). Emerging evidence indicates that mitophagy promotes recovery from radiation-induced injury (Ding et al. 2024; Jiao et al. 2024), thereby highlighting its potential as a therapeutic target after radiation exposure. Remarkably, studies further suggest that renal function is tightly regulated by mitophagy, with impaired or compromised mitophagy underlying numerous renal disorders (Fan et al. 2024; Wu et al. 2025; Yang et al. 2024a).

Mitophagy has a dual effect on apoptosis: it can preserve cells by eliminating damaged mitochondria and promote cell survival by upregulating anti-apoptotic proteins and downregulating pro-apoptotic proteins. This process helps stabilize the mitochondria and prevents the apoptotic cascade. However, overactivation of mitophagy may lead to mitochondrial depletion, disrupt energy production, and enhance apoptosis through boosting death signals. The overall impact of mitophagy depends on the extent of mitophagy activation and the specific molecules involved (Yang et al. 2024b). Therefore, preserving mitophagy function is regarded as a crucial therapeutic target after radiation exposure, promoting a shift in cellular responses from death pathways toward survival. Among the pathways identified to modulate mitophagy is activation of sirtuin proteins (SIRT1-7), a family of conserved protein NAD^+^-dependent deacetylases, along with their downstream target proteins, the forkhead box O (FOXO) transcription factors (Wan et al. 2022; Wang et al. 2025), emphasizing the therapeutic significance of agents capable of engaging this pathway.

Rosuvastatin (ROSU) is a commonly prescribed lipid-lowering medication that acts by hindering 3-hydroxy-3-methylglutaryl coenzyme A reductase, the rate-limiting enzyme in cholesterol formation. ROSU exhibits enhanced efficacy in optimizing lipid profiles relative to other statins (Cortese et al. 2016). In addition to its primary function of reducing low-density lipoprotein cholesterol, ROSU exhibits several pleiotropic effects, including anti-inflammatory and antioxidant properties (Fadel et al. 2025; Tang et al. 2024). Moreover, numerous experimental studies have extensively documented the protective effects of ROSU in different models of renal injury (Al-Ghanimi and Janabi 2025; Saad et al. 2024; Shafik et al. 2023). In particular, previous studies have reviewed the role of statins in autophagy activation (Liu et al. 2022; Mengual et al. 2022) and figured it via several ways, including (1) activation of SIRT1/FOXO3a molecular cascade, (2) AMP-activated protein kinase (AMPK) activation, and (3) accumulation of nuclear p53, which induces autophagy in a p53-dependent manner.

To the best of our knowledge, while ROSU demonstrates a therapeutic effect in various experimental models of renal injury, the influence of ROSU on nephrotoxicity linked to radiation exposure has not been investigated yet. Accordingly, the current study aims to elucidate the protective role of ROSU against acute and fractionated radiation-induced nephropathy, particularly emphasizing its role in the modulation of mitophagy response via the upstream SIRT1/FOXO3a signaling cascade.

## Material and methods

### Animals

Male Sprague Dawley (SD) rats (150–200 g) were used in the present study (*n* = 30). Animals were taken from the animal breeding facility of the National Centre for Radiation Research and Technology (NCRRT). They were acclimatized for at least 1 week before the experiment in the NCRRT animal facility under standard controlled conditions, including a temperature of 27 °C, constant relative humidity (45–50%), and a 12-h light/dark cycle. Rats were fed standard diet pellets containing a minimum of 5% fiber, 20% protein, 3.5% fat, 6.5% ash, and a vitamin blend, with ad libitum access to water. All animal experiments were conducted in accordance with the ARRIVE guidelines and in compliance with the U.K. Animals (Scientific Procedures) Act of 1986 and its associated guidelines, as well as EU Directive 2010/63/EU on the protection of animals used for scientific purposes. The study protocol and all experimental procedures were approved by the Research Ethics Committee of the National Centre for Radiation Research and Technology (REC-NCRRT), Cairo, Egypt (Permit Number: F/21A/25).

### Irradiation process

Radiation exposure was performed at the NCRRT employing the Gamma Cell-40 biological irradiator with a Caesium^137^ source (Atomic Energy of Canada Ltd, Sheridan Science and Technology Park, Mississauga, Ontario, Canada). Animals were exposed to whole-body gamma irradiation with a total dosage level of 8 Gray (Gy) (Zaher et al. 2023) either acute or fractionated (2 Gy/day for 4 days). The dosage rate during the experiment was 0.33 Gy/min. The animals were kept in cages with good ventilation and placed in a chamber 25 × 25 cm^2^ that was attached to the irradiation apparatus.

### Rosuvastatin preparation and administration

The ROSU was a kind gift sample from Hikma Pharmaceuticals Co. (6th October City, Egypt). It was dissolved in saline and given intraperitoneally at a dose of 10 mg/kg. The dose was selected based on previous studies concerned with its therapeutic effects in different experimental models of kidney injury (Jiang et al. 2020; Shafik et al. 2023). The intraperitoneal route was selected to ensure reliable dosing and consistent systemic exposure, as ROSU exhibits low oral bioavailability (approximately 20%) (Hanke et al. 2021).

### Experimental design

Male Sprague Dawley rats were randomly assigned into five groups (*n* = 6/group), and the protocol proceeded as follows:

- Normal control group (Normal): Rats received saline (5 ml/kg).
- Acute irradiated group (acute-IR): Rats received saline (5 ml/kg) and were exposed to acute whole-body gamma radiation (8 Gy) on the 4th day of the experiment.
- Treated/acute irradiated group (ROSU + acute-IR): Rats received ROSU (10 mg/kg, i.p.) and were exposed to acute whole-body gamma radiation (8 Gy) on the 4th day of the experiment.
- Fractionated irradiated group (Fr-IR): Rats received saline (5 ml/kg) and were exposed to fractionated whole-body gamma radiation for four consecutive days (2 Gy × 4) from the 3rd day of the experiment.
- Treated/fractionated irradiated group (ROSU + Fr-IR): Rats received rosuvastatin (10 mg/kg, i.p.) and were exposed to fractionated whole-body gamma radiation for four consecutive days (2 Gy × 4) from the 3rd day of the experiment.

**Fig. 1:**
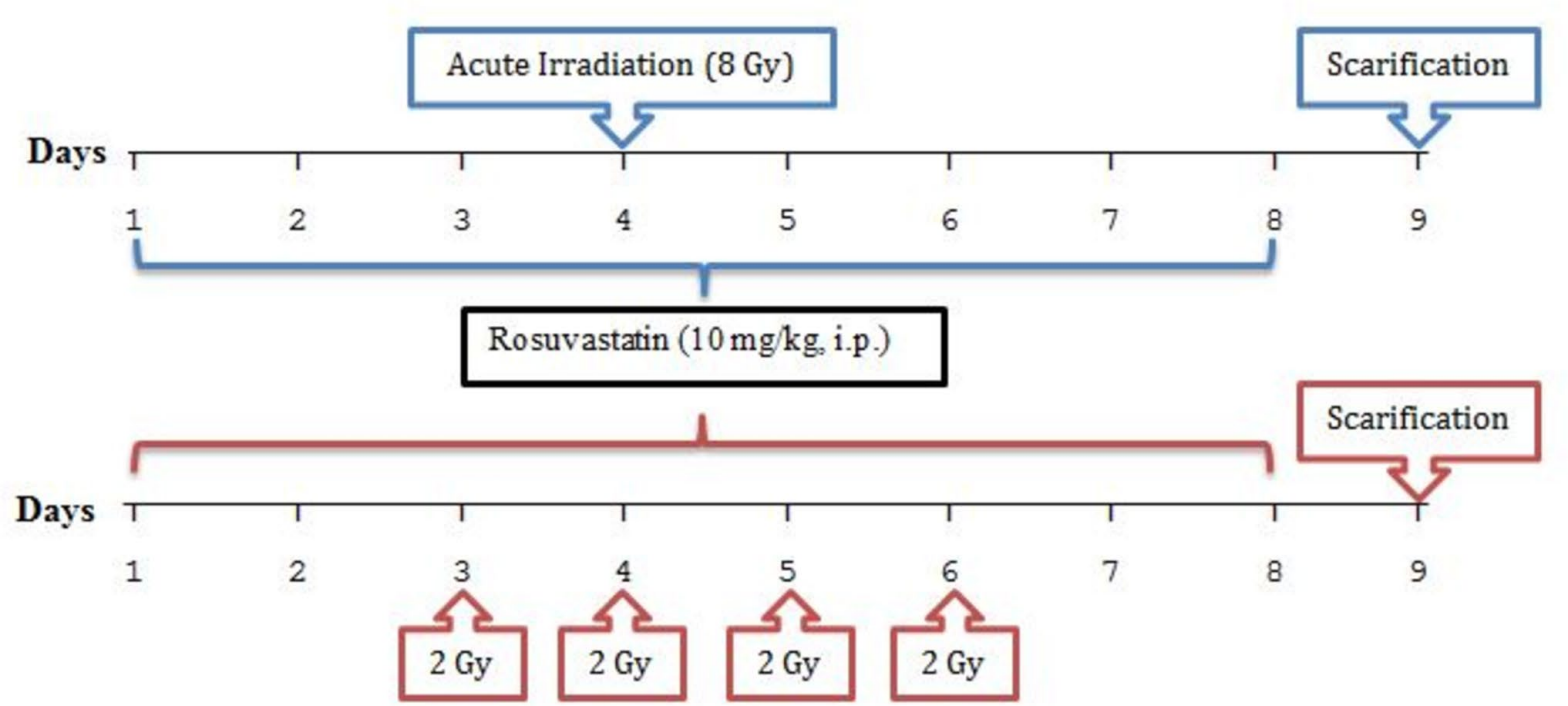
A workflow diagram depicting the experimental design

The experiment started on day 1 and extended for 9 days. Treatments, ROSU or saline, were intraperitoneally injected once daily for 8 days, starting from day 1. Groups 2 and 3 were exposed to acute whole body gamma radiation on day 4, while groups 4 and 5 were exposed to fractionated whole body gamma radiation on a daily basis starting from day 3 to day 6. At the end of the experiment (on the 9th day), rats were anesthetized with urethane (1.2 g/kg, i.p.) (Field et al. 1993) and scarified by cervical dislocation. A workflow diagram depicting the experimental design is presented in Fig. 1.

### Sample collection, tissue processing, and mitochondrial isolation

Blood samples were collected via decapitation in non-heparinized tubes, and serum was isolated by centrifugation at 1000 × *g* for 15 min for the estimation of kidney functions. The kidneys were carefully dissected, rinsed with ice-cold saline, and portioned for different analyses: some portions were used to prepare total tissue lysate, others for mitochondrial fraction isolation, and the remaining portions were fixed in 10% formalin for histopathological and immunohistochemical examinations.

For the preparation of total tissue lysate, kidney portions were homogenized in phosphate-buffered saline (pH 7.4) using an Ultra-Turrax tissue homogenizer (IKA, Staufen, Germany) to obtain a 20% (w/v) homogenate. The homogenate was centrifuged at 1000 × g for 15 min, and the supernatant was stored at − 80 °C for biochemical analyses.

For mitochondrial fraction analysis, mitochondria were isolated using a differential centrifugation procedure as described by Fernández-Vizarra et al. (2002) for subsequent measurement of mitophagy biomarkers. Briefly, the kidneys were homogenized in a cold isotonic buffer. The homogenate was centrifuged 3 times at 1000 × *g* for 10 min at 4 °C to precipitate tissue fragments and nuclei to produce mitochondria-rich supernatant. Finally, the mitochondria were isolated after centrifugation at 12,000 × *g* for 10 min at 4 °C.

Protein levels were determined using a commercial bicinchoninic acid (BCA) protein assay kit (Cat# 23,225, Thermo Fisher Scientific, Waltham, MA, USA), following the manufacturer’s protocol.

### Colorimetric assessment

Kidney functions were evaluated by measuring blood urea nitrogen (BUN) and serum creatinine levels, using a commercial colorimetric urea kit (Cat# UR 2110) and a creatinine kit (Cat# CR 1251), purchased from Biodiagnostics (Giza, Egypt), and following the manufacturer’s instructions. The values were given as mg/dl.

Renal oxidative imbalance biomarkers were assessed using colorimetric assay kits for malondialdehyde (MDA; Cat# MD 2529), superoxide dismutase (SOD; Cat# SD 2521), reduced glutathione (GSH, Cat# GR 2511), and glutathione peroxidase (GPx, Cat# GP 2524), all obtained from Biodiagnostics (Giza, Egypt), and performed according to the manufacturer’s protocols. The results were expressed as follows: MDA in nmol/mg protein, SOD in U/mg protein, GSH in mmol/mg protein, and GPx in U/mg protein.

### Histopathological examination of kidney tissues

Kidney tissues were sectioned and fixed immediately in 10% neutral-buffered formalin for 24 h. The fixed specimens were then trimmed, washed, and dehydrated with alcohol, cleared in xylene, and embedded in paraffin using a tissue processing machine. For sliding, a section of 4–6 μm thickness was used. Hematoxylin and eosin (H&E) stains were applied to the prepared slides and examined under a compound microscope. The grading system for renal lesions was done according to Zhang et al. (2008). Slides were coded, and sections were randomly selected from each kidney to ensure representative sampling and minimize bias. Evaluation of the kidney tissue specimen was carried out by two independent observers who were blinded to the experimental protocol.

### Real-time quantitative polymerase chain reaction (RT-qPCR)

Total RNA from cells was extracted using the Trizol reagent kit (Cat# R2072, ZYMO RESEARCH CORP. USA), and then the quantity and quality were assessed by the Beckman dual spectrophotometer (USA). The SuperScript IV One-Step RT-PCR kit (Cat# 12,594,100, Thermo Fisher Scientific, Waltham, MA, USA) was utilized for reverse transcription of extracted RNA, followed by PCR in one step. GAPDH was used as the internal control, and each RT-qPCR experiment included three technical replicates. After the RT-PCR run, the data were expressed in cycle threshold (Ct). The PCR data sheet includes Ct values of the assessed genes (SIRT1 and FOXO3a) versus the corresponding housekeeping gene (GAPDH). The relative expression of target genes was calculated using the 2^−∆∆Ct^ formula (Pfaffl 2001). Primer sequences are detailed in Table 1.

**Table 1:** Primer sequence of target genes

	Forward sequence	Reverse sequence
SIRT1	TAGACACGCTGGAACAGGTTGC	CTCCTCGTACAGCTTCACAGTC
FOXO3a	TACGAGTGGATGGTGCGCTG	AGGTTGTGGCGGATGGAGTTC
GAPDH	TGGATTTGGACGCATTGGTC	TTTGCACTGGTACGTGTTGAT

*SIRT1*, Sirtuin 1; *FOXO3a*, Forkhead Box O3a; *GAPDH*, glyceraldehyde 3-phosphate dehydrogenase

### Enzyme-linked immunosorbent assay (ELISA)

The ELISA technique was carried out using rat-specific kits to detect the levels of autophagy-related gene 5 (ATG5) (Cat# MBS2709569), PINK1 (Cat# MBS9343426), Parkin (Cat# MBS722554), and FOXO3a (Cat# MBS9305817), all purchased from Mybiosource (San Diego, CA, USA) and followed the manufacturer’s instructions. Results are expressed as follows: ATG5 and PINK1 in ng/mg protein, while Parkin and FOXO3a in pg/mg protein.

### Immunohistochemical (IHC) assay

The IHC analysis was used for measuring SIRT1 and cleaved caspase‐3 protein expression in kidney tissues. Paraffin-embedded kidney tissue sections of 3 μm thickness were rehydrated first in xylene, followed by graded ethanol solutions. Afterwards, the slides were blocked for 2 h using 5% bovine serum albumin (BSA) in Tris-buffered saline (TBS). Then, IHC was performed using a standard streptavidin–biotin-peroxidase procedure. Sections from all groups were incubated with rabbit polyclonal anti-cleaved caspase-3 (Cat# GB11532, ServiceBio Technology Co., Ltd, Wuhan, China) or rabbit polyclonal anti-SIRT1 (Cat# GB11171, ServiceBio Technology Co., Ltd, Wuhan, China). Next, rinsing was done thoroughly with TBS, and sections were incubated with a biotinylated goat anti-rabbit secondary antibody. This was followed by washing and then 30 min of incubation with horseradish-peroxidase-conjugated streptavidin solution. Finally, the sections were washed with TBS and visualized with 3,3′-diaminobenzidine containing 0.01% H_2_O_2_. The protein expression of SIRT1 and cleaved caspase-3 was detected by measuring the intensity of the developed brown color using a Leica application for slide analysis (Leica Biosystems, Germany). The quantitative analysis was carried out by counting the number of positive cells in 3 high microscopic power fields (× 400), each one representing a different rat.

### Statistical analysis

Data normality was verified by the Shapiro–Wilk test, and homogeneity of variance was ensured using Bartlett’s test. Data were screened for outliers using *z*-score analysis. Continuous variables will be presented as the mean ± standard deviation (SD). Intergroup differences will be analyzed using one-way analysis of variance (ANOVA) followed by Tukey’s as a post-hoc test. The probability level less than 0.05 (*p* < 0.05) is considered statistically significant. Statistical tests and figure constructions were carried out using GraphPad Prism® software (version 5, San Diego, CA, USA).

## Results

### Rosuvastatin alleviated renal dysfunction in acute and fractionated radiation-treated rats

**Fig. 2:**
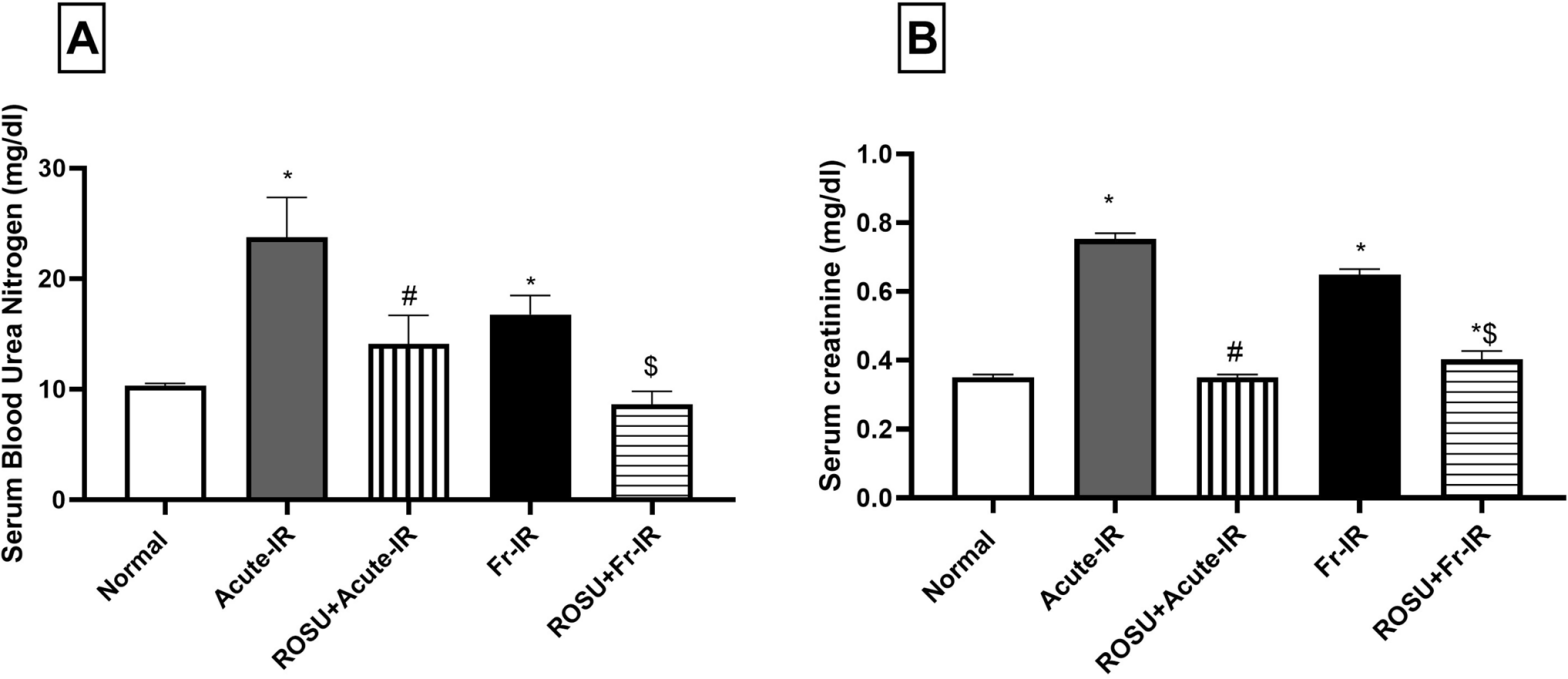
Effect of rosuvastatin (ROSU, 10 mg/kg, i.p.) on kidney functions in rats exposed to acute (8 Gy) or fractionated (2 Gy × 4) radiation. **A** BUN level and **B** Serum creatinine level. Data are presented as mean ± SD (*n* = 6). Statistical analysis was conducted using one-way ANOVA, followed by *Tukey’s post-hoc* test at *p* < 0.05, as compared with (*) normal, (#) acute-IR, and ($) Fr-IR groups. Acute-IR, acute irradiation; BUN, blood urea nitrogen; FR-IR, fractionated irradiation

As depicted in Fig. 2, exposure to acute radiation resulted in renal injury and impairment of renal functions, shown by a 2.3-fold increase in (A) BUN and a 2.1-fold increase in (B) serum creatinine, compared to the normal group. However, pretreatment with ROSU (10 mg/kg) conferred renal protection, demonstrated by a 40.5% reduction in BUN and a 53.5% decrease in serum creatinine, relative to the acute radiation group.

Similarly, rats subjected to fractionated radiation exhibited an elevation in BUN and serum creatinine, reaching 1.6- and 1.9-fold, respectively, in comparison to the normal group. Nonetheless, pretreatment with ROSU diminished these elevated kidney functions, reducing BUN by 48.5% and decreasing serum creatinine by 38%, relative to the fractionated radiation group.

### Rosuvastatin ameliorated renal injury in acute and fractionated radiation-treated rats

**Fig. 3:**
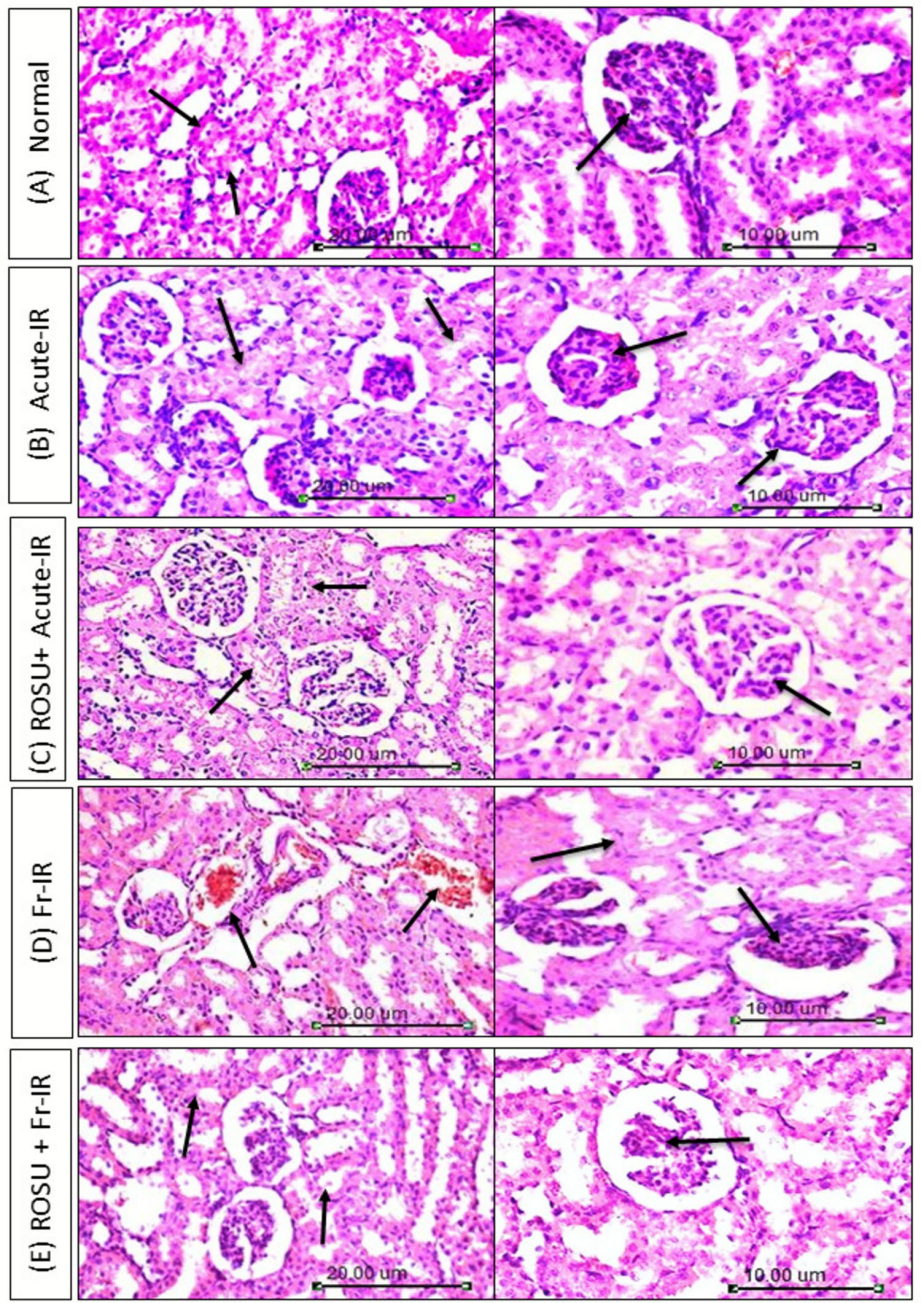
Photomicrographs of H&E-stained kidney tissue sections from rats exposed to acute (8 Gy) or fractionated (2 Gy × 4) radiation and pretreated with rosuvastatin (ROSU, 10 mg/kg, i.p.). Arrows in the first column (× 200) indicate the renal tubular epithelium histology across different groups, showing intact epithelium in the normal group, epithelial swelling in the irradiated groups, and mild epithelial swelling in the treated groups. Arrows in the second column (× 400) illustrate the histological architecture of the glomeruli, showing normal histological structure of the glomerulus in the normal group, shrinkage of capillary tufts and widening of Bowman’s space of some glomeruli in irradiated groups, and mild shrinkage of capillary tufts in treated groups. Acute-IR, acute irradiation; FR-IR, fractionated irradiation

The kidney tissue section of the normal control group showed normal histological structure of renal parenchyma characterized by circumscribed glomeruli with intact capillary tufts and Bowman’s capsule. The renal tubules of both proximal and distal convoluted tubules showed normal organization of epithelial lining (score 0) (Fig. 3A).

In the acute irradiation model (Fig. 3B), kidney tissue of irradiated rats exhibited shrinkage of capillary tufts and widening of Bowman’s space in some glomeruli. The renal tubules showed marked swelling of the tubular epithelial lining accompanied by narrowing and occlusion of the tubular lumen by albuminous and cellular casts. Some proximal convoluted tubules showed nuclear pyknosis of their tubular epithelial lining. Cellular necrosis and apoptosis < 50% (score 3) were seen. On the other side, irradiated rats treated with ROSU exhibited mild shrinkage of capillary tufts with widening of Bowman’s space in some glomeruli. Degeneration of renal tubular epithelial lining appeared in the form of swelling and granularity of its cytoplasm with intra-tubular albuminous casts. Few numbers of apoptotic cells were seen in between tubular epithelial lining. Tubular epithelial cell necrosis and apoptosis < 25% were seen (score 2) (Fig. 3C).

With respect to the fractionated irradiation group (Fig. 3D), renal tissue section revealed shrinkage of capillary tufts with widening of Bowman’s space, which contained eosinophilic proteinaceous material. Congestion of peri-tubular blood capillaries was also noticed. Degeneration of renal tubular epithelial lining appeared in the form of swelling, with intratubular albuminous casts being observed. Tubular epithelial cell necrosis and apoptosis < 75% were seen (score 4). On the contrary, rats exposed to fractionated radiation and treated with ROSU demonstrated marked improvement in comparison with its corresponding control. Mild shrinkage of capillary tufts with widening of Bowman’s space and mild swelling of tubular epithelial cell lining without significant necrosis or apoptosis was seen (score 1) (Fig. 3E).

### Rosuvastatin modulated renal oxidative imbalance in acute and fractionated radiation-treated rats

**Fig. 4:**
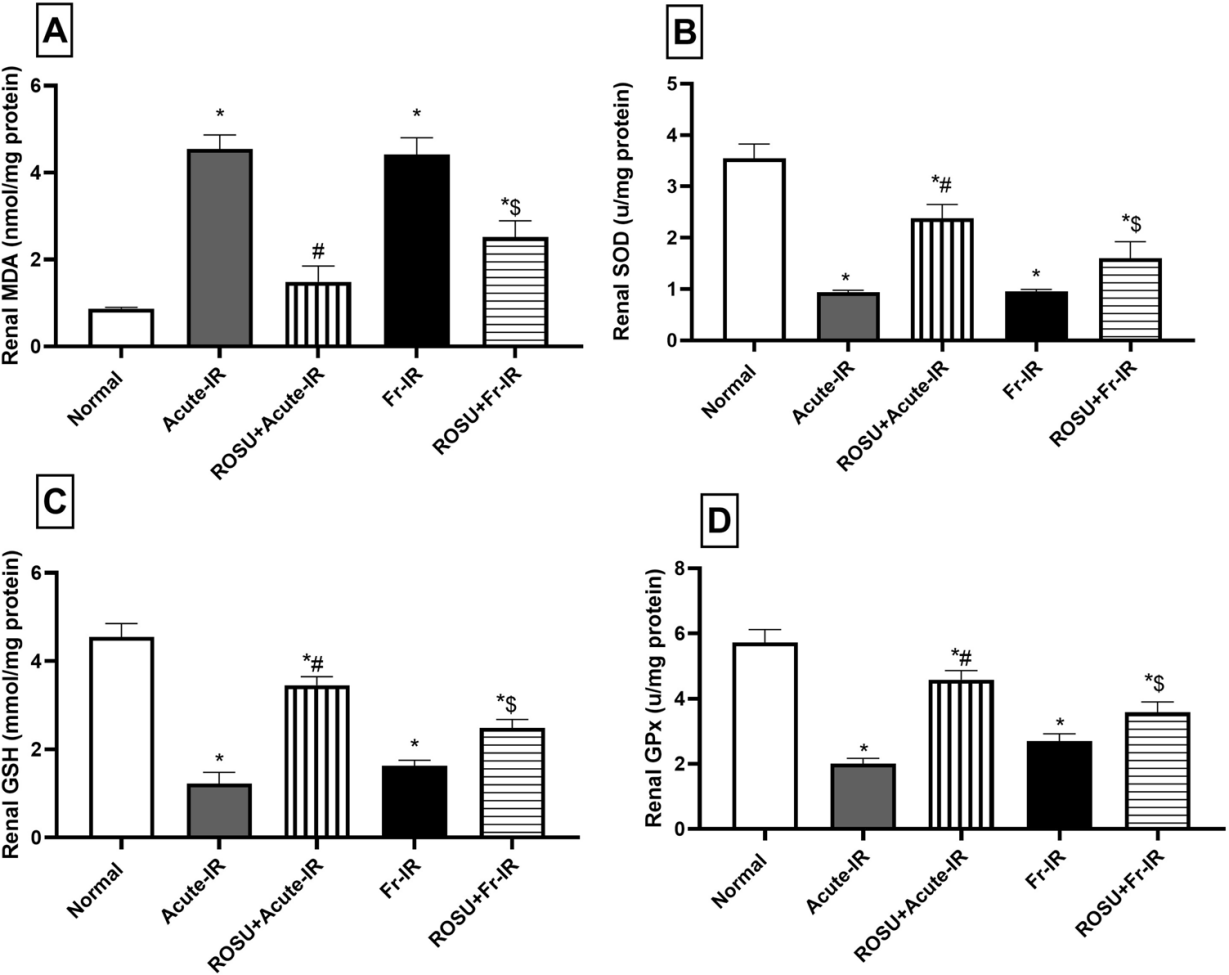
Effect of rosuvastatin (ROSU, 10 mg/kg, i.p.) on renal oxidative stress biomarkers in rats exposed to acute (8 Gy) or fractionated (2 Gy × 4) radiation. **A** Malondialdehyde (MDA) content; **B** Superoxide dismutase (SOD) activity; **C** Reduced glutathione (GSH) content; and **D** Glutathione peroxidase (GPx) activity. Data are presented as mean ± SD (*n* = 6). Statistical analysis was conducted using one-way ANOVA, followed by *Tukey’s post-hoc* test at *p* < 0.05, as compared with (*) normal, (#) acute-IR, and ($) Fr-IR groups. Acute-IR, acute irradiation; FR-IR, fractionated irradiation

As shown in Fig. 4, exposure to acute or fractionated radiation induced a marked oxidative imbalance. In the acute and fractionated groups, respectively, (A) MDA level increased by 5.2- and 5.1-fold, while (B) SOD activity declined by 72.8% and 72.3%, relative to normal. This was accompanied by a notable reduction in (C) GSH level by 73.2% and 64.2%, along with a substantial decrease in (D) GPx activity by 64.9% and 52.8%, compared with the normal group.

On the other hand, as compared to the acute radiation group, pretreatment with ROSU reduced the MDA level by 67.3%, while elevated the SOD activity, GSH level, and GPx activity by 152.9%, 183%, and 127.7%, respectively. In comparison to the fractionated radiation group, ROSU pretreatment led to a reduction in MDA level by 43% and improved the antioxidant defenses, demonstrated by a 1.7-fold rise in SOD activity, a 1.5-fold increase in GSH level, and a 1.3-fold increase in GPx activity.

### Rosuvastatin enhanced renal SIRT1/FOXO3a in acute and fractionated radiation-treated rats

**Fig. 5:**
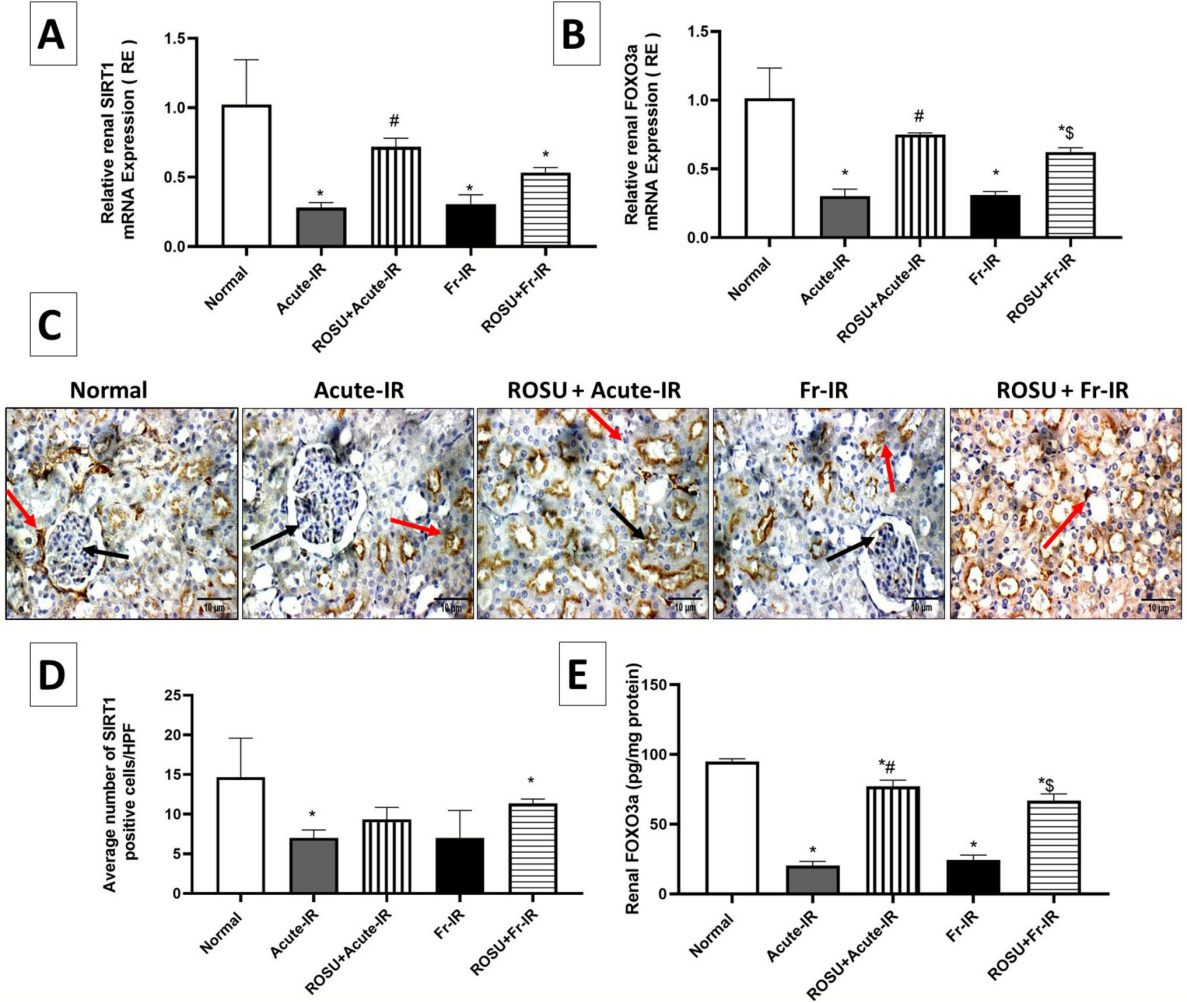
Effect of ROSU (10 mg/kg, i.p.) on renal SIRT1/FOXO3a expression in rats exposed to acute (8 Gy) or fractionated (2 Gy × 4) radiation. **A** mRNA levels of SIRT1; **B** mRNA levels of FOXO3a; **C** Representative photomicrographs of immunohistochemical examination of SIRT1 protein. Normal and treated sections displayed high SIRT1 protein expression indicated by intense brown staining, while irradiated groups displayed low SIRT1 expression, indicated by less brown staining. The black arrow showed the positive cells in glomeruli, while the red arrow showed those in tubules; **D** Quantitative analysis of SIRT1 was done by counting the number of positive cells in three high microscopic power fields (HPF  × 400), each one represents a different rat; and **E** FOXO3a protein level. Data are presented as mean ± SD (*n* = 6). Statistical analysis was conducted using one-way ANOVA, followed by *Tukey’s post-hoc* test at *p* < 0.05, as compared with (*) normal, (^**#**^) acute-IR, and ($) Fr-IR groups. Acute-IR, acute irradiation; FOXO3a, forkhead box O3a; FR-IR, fractionated irradiation; SIRT1, sirtuin1

As shown in Fig. 5, qRT-PCR was performed to evaluate the expression levels of (A) SIRT1 and (B) FOXO3a genes in renal tissue. Rats exposed to acute radiation (8 Gy) exhibited a significant decrease in renal gene expression of SIRT1 and FOXO3a, declining by 72.6% and 70.1%, respectively, compared to the normal group. Pretreatment with ROSU increased SIRT1 gene expression by 2.6-fold and FOXO3a gene expression by 2.5-fold, relative to the acute irradiation group. Exposure to fractionated radiation resulted in a substantial drop in the renal gene expression of SIRT1 by 70.2% with a consequent decrease in the FOXO3a gene expression by 69.4%, relative to the normal group. Nevertheless, pretreatment with ROSU slightly elevated SIRT1 gene expression and significantly increased FOXO3a gene expression, reaching 1.7- and twofold, respectively, compared to the fractionated irradiation group.

The protein level of SIRT1 was validated by the IHC technique (Fig. 5C). Compared with the normal group, acute and fractionated irradiation markedly suppressed renal SIRT1 protein expression, with quantitative analysis confirming reductions by 52.3% and 52.3%, respectively (Fig. 5D). Conversely, treatment with ROSU improved these alterations, increasing SIRT1 immunostaining by 33.3% in the acute irradiation model and by 61.9% in the fractionated irradiation model, compared to their respective irradiated controls. Although the differences between the irradiated groups and their corresponding treatment groups did not reach statistical significance, the magnitude of change was considerable, as reflected by the large effect size in the acute irradiation model (Cohen’s *d* = 1.80) and in the fractionated irradiation model (Cohen’s *d* = 1.74), indicating a substantial improvement in the treated animals.

Furthermore, the protein expression of FOXO3a in renal tissue was assessed using ELISA to confirm the changes observed at the gene level (Fig. 5E). Exposure to acute or fractionated radiation significantly reduced renal FOXO3a protein levels by 78.6% and 74.4%, respectively, relative to the normal group. Notably, pretreatment with ROSU effectively counteracted these radiation-induced reductions, where ROSU increased FOXO3a protein expression by 279.7% in the acute irradiation model and by 174.8% in the fractionated irradiation model, relative to their respective irradiated controls.

These results demonstrate that ROSU can substantially restore SIRT1 and FOXO3a protein expression in renal tissue, highlighting its renoprotective effect in mitigating radiation-induced renal damage.

### Rosuvastatin boosted renal mitophagy biomarkers in acute and fractionated radiation-treated rats

**Fig. 6:**
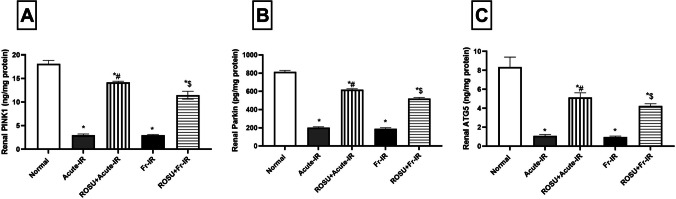
Effect of rosuvastatin (ROSU, 10 mg/kg, i.p.) on renal mitophagy biomarkers in rats exposed to acute (8 Gy) or fractionated (2 Gy × 4**)** radiation. **A** PTEN-induced putative kinase protein 1 (PINK1) level; **B** Parkinson protein 2 E3 ubiquitin protein ligase (Parkin) level; **and C** Autophagy-related gene 5 (ATG5) level. Data are presented as mean ± SD (*n* = 6). Statistical analysis was conducted using one-way ANOVA, followed by *Tukey’s post-hoc* test at *p* < 0.05, as compared with (*) normal, (#) acute-IR, and ($) Fr-IR groups. Acute-IR, acute irradiation; ATG5, autophagy-related 5; FR-IR, fractionated irradiation

As illustrated in Fig. 6, acute irradiation caused a pronounced suppression of the mitophagy machinery, reflected by significant decreases in renal (A) PINK1, (B) Parkin, and (C) ATG5 protein levels by 83.7%, 75.3%, and 87%, respectively, compared with the normal group. Pretreatment with ROSU effectively counteracted these reductions, markedly elevating PINK1, Parkin, and ATG5 levels by 379%, 207.1%, and 373.2%, respectively, relative to the acute irradiation group.

A similar pattern was observed following fractionated irradiation, which led to substantial declines in PINK1, Parkin, and ATG5 protein levels by 83.7%, 76.7%, and 88.6%, respectively, compared with normal rats. In contrast, ROSU administration significantly enhanced the mitophagy response, increasing renal PINK1, Parkin, and ATG5 expression by 288.3%, 173.7%, and 344.6%, respectively, relative to the fractionated irradiation group.

### Rosuvastatin suppressed renal cell apoptosis in acute and fractionated radiation-treated rats

**Fig. 7:**
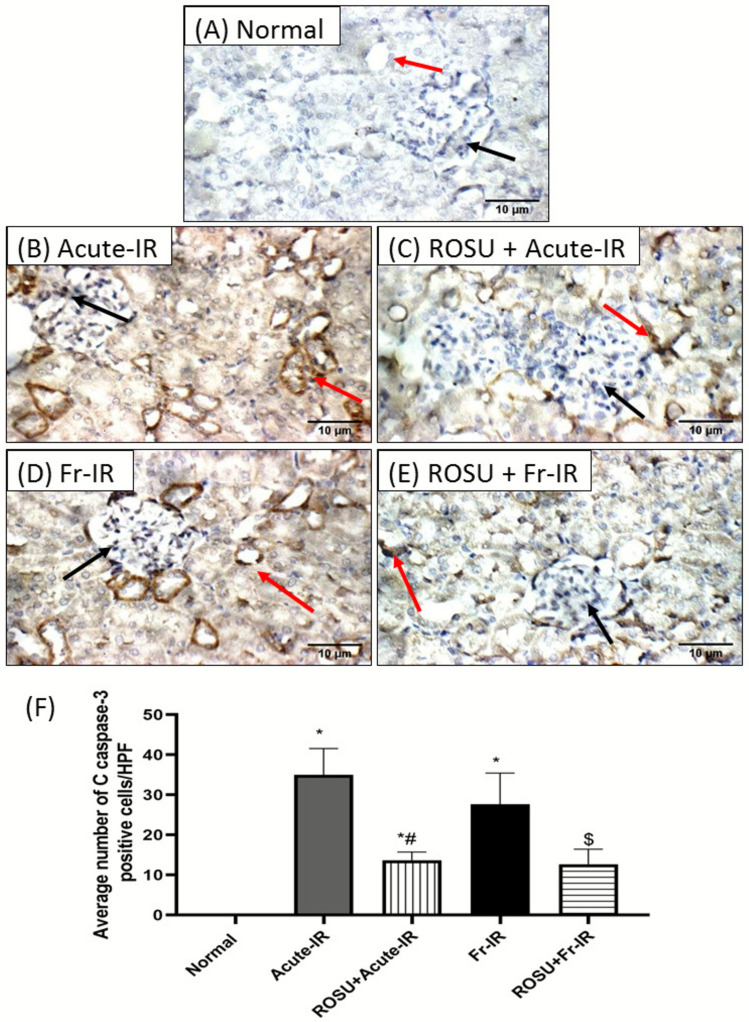
Immunohistochemical examination of renal cleaved caspase-3 in rats exposed to acute (8 Gy) or fractionated (2 Gy × 4) radiation and pretreated with rosuvastatin (ROSU, 10 mg/kg, i.p.). **A**–**E** Representative sections displayed minimal cleaved caspase-3 expression in the normal group, high cleaved caspase-3 expression, indicated by intense brown staining in the irradiated groups, and low cleaved caspase-3 expression, showing faint cytoplasmic staining in the treated groups. The black arrow showed the positive cells in glomeruli, while the red arrow showed those in tubules. **F** Quantitative analysis of cleaved caspase-3 was done by counting the number of positive cells in three high microscopic power fields (HPF × 400), each representing a different rat. Data are presented as mean ± SD (*n* = 3). Statistical analysis was conducted using one-way ANOVA, followed by *Tukey’s post-hoc* test at *p* < 0.05, as compared with (*) normal, (#) acute-IR, and ($) Fr-IR groups. Acute-IR, acute irradiation; C caspase, cleaved caspase; FR-IR, fractionated irradiation

The expression of cleaved caspase-3 protein was estimated in renal tissues by IHC (Fig. 7). A negative expression was detected in the kidney tissues from the normal group, indicating the absence of apoptotic activation under physiological conditions. In contrast, both acute and fractionated irradiation triggered a pronounced increase in cleaved caspase-3 immunostaining within the renal glomeruli and tubular epithelial cells, reflecting substantial activation of the apoptotic cascade in response to radiation exposure. Notably, ROSU administration significantly attenuated the radiation-induced apoptotic response which is further confirmed by quantitative analysis. In the acute irradiation model, treatment with ROSU reduced cleaved caspase-3 expression by 61%, while in the fractionated irradiation model, ROSU treatment produced a 54.2% reduction in the protein expression, compared with their respective irradiated controls. These findings highlight the potent anti-apoptotic effect of ROSU in mitigating radiation-induced renal injury.

## Discussion

Selective targeting of cancerous tissue presents a significant challenge in radiotherapy, as toxicity to healthy tissue is a major adverse effect that restricts radiotherapy application and leads to inadequate tumor control (Verginadis et al. 2025). As a matter of fact, radiation exposure diminishes mitochondrial activity, resulting in the overproduction and buildup of mitochondrial ROS, which leads to malfunction and ultimately leads to cell death (Rong et al. 2025). Hence, mitigating mitochondrial harm served as a potential target for most therapeutics. Indeed, ROSU has previously shown beneficial effects in various kidney injury models; nevertheless, its potential radioprotective role in mitigating renal injury, triggered by acute and fractioned irradiation exposure, has not yet been defined. Accordingly, the current study aimed to assess the beneficial impact of ROSU against radiation-induced nephropathy with a deep mechanistic insight into its role in restoring mitophagy machinery and hindering the intrinsic apoptotic cascade.

First, rats were exposed to whole-body radiation at a dose level of 8 Gy, either acute or fractionated. Radiation-induced nephropathy was initially evaluated through histopathological examinations, which demonstrated a more pronounced degeneration of the renal tubular epithelial lining, along with increased tubular epithelial cell necrosis and apoptosis in the fractionated radiation group compared to the group subjected to acute radiation. This result aligned with a prior study of El Bakary et al. (2022), confirming a greater damage score in the renal tissue of rats subjected to fractionated radiation compared to acute irradiation exposure. In practice, such an outcome was unexpected, as it had been previously proven that fractionation dosage facilitates the repair of sub-lethal damage, repopulation, reoxygenation, and reassortment of cells during the cell cycle, hence permitting greater total dosages (Schaue and McBride 2015). These histopathological alterations are well-correlated with the observed impairment in renal function, evidenced by increased serum creatinine and BUN following radiation exposure (Hamed et al. 2024). Radiation-triggered apoptosis or necrosis of renal cells reduces nephron number and compromises glomerular and tubular integrity, leading to reduced filtration and subsequent accumulation of nitrogenous wastes, including urea and creatinine, in the serum (Ahmad et al. 2023).

The initial cascade of radiation-induced tissue harm may occur by direct destruction of chemical bonds and electron displacement, resulting in double-strand breaks of DNA, or indirectly through overproduction of ROS, which are primarily produced by mitochondrial oxidative metabolism or ionized water. When ROS surpasses the antioxidant capacity, a state of oxidative stress ensues which further leads to oxidation of cellular macromolecules, e.g., lipids, proteins, or DNA (Wei et al. 2019). Overproduction of ROS is thought to be a major factor in the disruption of normal cell function and subsequently leads to tissue damage. As demonstrated in the current study, the oxidative stress status was defined by the rise in the renal MDA, lipid peroxidation end product, alongside depletion of antioxidant defenses, including SOD, GSH, and GPx. Thus, radiation-induced ROS production likely underlies the observed histopathological renal tissue damage and impaired kidney function. Hence, controlling the oxidative stress was considered to play a key role in hindering the radiation-induced normal tissue injury (Saadat et al. 2023; Soliman et al. 2019). Notably, ROSU administration in both acute and fractionated radiation models effectively reversed the histopathological alterations, improved renal function, and reestablished oxidative balance, likely through attenuation of ROS-mediated damage. That was in line with previous studies that clearly demonstrated the antioxidant potential of ROSU in radiation-induced spleen and cardiac tissues injury (Fadel et al. 2025; Fahim et al. 2023).

Beyond its antioxidant potential, the present study further elucidates the mechanisms underlying ROSU’s protective effects by highlighting its ability to modulate the mitophagy response under different radiation regimens. Mitochondria normally respond to cellular insults by undergoing fission or activating mitophagy, a selective form of autophagy that may precisely remove damaged mitochondria to maintain mitochondrial homeostasis and cell survival (Onishi et al. 2021). Mitophagy induction is mechanistically dependent on the mitochondrial priming PINK1/Parkin pathway that works in tandem with the autophagy machinery (Ashrafi and Schwarz 2013). Under basal conditions, PINK1 is maintained at low abundance due to rapid degradation by mitochondrial inner membrane-associated proteases. Upon mitochondrial stress and loss of membrane potential, PINK1 accumulates on the outer mitochondrial membrane, where it undergoes dimerization and autophosphorylation. This activation facilitates the recruitment and phosphorylation of Parkin, which ubiquitinates outer mitochondrial membrane proteins and recruits mitophagy receptors that interact with LC3 on the forming phagophore. The phagophore then expands, with the involvement of ATG proteins such as ATG5, to enclose the impaired mitochondrion within a double-membrane autophagosome, which ultimately fuses with lysosomes to degrade the damaged organelle (Nguyen et al. 2016).

Indeed, radiation exposure markedly increases mitochondrial ROS production, which induces pore formation and disrupts the mitochondrial membrane potential, ultimately resulting in mitochondrial dysfunction (Li et al. 2019). Although this condition would typically be expected to promote mitophagy, our findings revealed that radiation exposure unexpectedly altered the cellular response, resulting in suppressed mitophagy—as evidenced by the decreased levels of PINK, Parkin, and ATG5—and a concomitant activation of apoptosis, indicated by the increased expression of cleaved caspase-3. Noteworthy, the reaction of mitochondria toward radiation may fluctuate according to the start, dosage, and the time gap between exposure and scarification (Averbeck and Rodriguez-Lafrasse 2021). There is a complex interplay between mitophagy and apoptosis. During exposure to moderate levels of ROS, mitochondria can activate mitophagy, promoting survival by removing damaged mitochondria; however, under conditions of overwhelming mitochondrial damage, the mitochondrial outer membrane permeability is altered, and damaged mitochondria are not cleared efficiently due to inactivation of mitophagy pathway proteins, prompting apoptosis to become prevalent (Lin et al. 2024; Yang et al. 2024b). Apoptosis is primarily driven by the mitochondria-dependent release of cytochrome c, which activates caspases and initiates the cell death cascade. Once released into the cytosol, cytochrome c binds apoptotic protease-activating factor-1, leading to the activation of procaspase-9 and subsequently caspase-3, the key executor of apoptosis (Lin et al. 2024; Yang et al. 2024b). This perspective may account for the reduced level of mitophagy-related proteins alongside the heightened caspase-3 expression observed either after acute or fractionated radiation exposure. Our findings align with those of He et al. (2025), who reported suppressed mitophagy accompanied by reduced PINK/Parkin protein levels in the colonic tissues of irradiated rats. In addition, the study of Fadel et al. (2025) manifested the elevated content of caspase-3 in cardiac tissue of irradiated rats, which was reversed by ROSU administration. Conversely, other studies have reported differing findings, indicating that radiation exposure can upregulate mitophagy biomarkers (Ding et al. 2024; Zhang et al. 2025).

Agreeing with the current status induced by radiation exposure in our study, a wide array of studies have revealed that mitophagic deficiency significantly increased tubular cell apoptosis and tissue damage, while activation of mitophagic proteins plays a vital role in maintaining mitochondrial function and reducing apoptosis of the renal tubular cells (Bhatia and Choi 2023; Duan et al. 2022; Gao et al. 2020; Lin et al. 2021), underscoring the value of targeting this pathway therapeutically. As shown in the current data, ROSU administration upregulated the mitophagy protein biomarkers, PINK1, Parkin, and ATG5, while suppressing the caspase-3 apoptotic biomarker, thus shifting the cellular response to a survival mechanism. To some extent, the role of ROSU in modulating mitophagy machinery during radiation exposure has not been elucidated in previous studies. However, some studies have explored the underlying mechanisms of different statins toward mitophagy activation. Statins mitigate mitochondrial ROS and enhance mitochondrial quality-control processes, thereby creating a cellular environment that favors activation of the PINK1/Parkin mitophagy pathway (Mollazadeh et al. 2021). Moreover, Andres et al. (2014) investigated the cardioprotective effect of *simvastatin* and attributed its effect to the depletion of Coenzyme Q10, a downstream product of the mevalonate pathway. This depletion impairs mitochondrial electron transport, resulting in loss of membrane potential and triggering mitophagy. In addition, *Pitavastatin* was reported to protect the endothelial progenitor cell via calcium/calmodulin-dependent protein kinase that promoted mitochondrial calcium release and induced phosphorylation of PINK1/Parkin, leading to activation of mitophagy (Yang et al. 2022).

Given the lack of studies clarifying ROSU’s mechanism in mitophagy regulation—and with existing evidence addressing only its role in autophagy activation through pathways such as SIRT/FOXO—the present investigation focused on the SIRT1/FOXO3a axis, recognizing it as a crucial intermediary in the molecular cascade underlying ROSU-mediated mitophagy activation (Liu et al. 2022; Wan et al. 2022). SIRT1, primarily located in the nucleus, regulates key cellular processes—including oxidative stress response, energy metabolism, aging, and apoptosis—by deacetylating target proteins such as the tumor suppressor protein p53, peroxisome proliferator-activated receptor-alpha, and members of the FOXO family (Chang and Guarente 2014). Among these, FOXO3a plays a central role in maintaining redox homeostasis (Kobayashi et al. 2005). Through SIRT1-mediated deacetylation, FOXO3a enhances the transcription of antioxidant enzymes, notably SOD, which supports cellular defense against oxidative stress and modulates apoptosis (Kops et al. 2002).

Remarkably, the current investigation clearly demonstrated that radiation exposure, whether acute or fractionated, significantly downregulated the gene and protein expression of SIRT1/FOXO3a. Exposure to ionizing radiation leads to extensive oxidative and inflammatory injury that directly impacts the SIRT1/FOXO3a axis. Radiation-induced ROS overproduction depletes NAD^+^ stores—essential for SIRT1 activity—and promotes oxidative modification of the SIRT1 protein, collectively suppressing its transcription and enzymatic function (Lewis et al. 2019; Yue et al. 2025). Concurrently, activation of the renal DNA damage response enhances p53 activity, a known transcriptional repressor of SIRT1, further lowering its expression following radiation exposure (Qin et al. 2021a). Radiation also promotes renal inflammation, particularly via nuclear factor-kappa B (NF-κB), which antagonizes SIRT1 transcriptional activity (Zhang et al. 2024). Consequently, impaired SIRT1 further reduces FOXO3a deacetylation and activation, weakening its antioxidant regulatory function. Hence, activation of the SIRT1/FOXO3a has been proposed as a potential target for improving radiation-induced damage (Qin et al. 2021b).

Crucially, pretreatment with ROSU showed a pronounced elevation in renal SIRT1/ FOXO3a gene and protein expression levels. Our results are supported by prior studies that highlighted the cardioprotective effect of ROSU through activating the SIRT1/FOXO3a pathway (Khayatan et al. 2022; Ren et al. 2022). Mechanistically, ROSU may elevate SIRT1 levels by activating AMPK, which increases intracellular NAD^+^ availability, thereby enhancing SIRT1 expression and deacetylase activity (Lai et al. 2021). Moreover, the stimulatory effect of ROSU on SIRT1 may result from its ability to reduce ROS and oxidative stress, thereby relieving ROS-mediated suppression of SIRT1, as well as from inhibition of the NF-κB pathway, which otherwise downregulates SIRT1 expression under inflammatory conditions (Ren et al. 2021). Interestingly, SIRT1 promotes mitophagy by deacetylating FOXO3a, which upregulates Rab7 expression, a small GTP-binding protein that facilitates autophagosome-lysosome fusion, thereby increasing mitophagy flux (Wan et al. 2022), and contributing to the restoration of mitophagy-related proteins.

From a translational perspective, the present findings suggest that ROSU may protect renal cells by modulating mitophagy in the context of radiation exposure. While this study was conducted in a rat model, the results provide a mechanistic rationale for exploring ROSU as a nephroprotective agent in patients undergoing radiotherapy for lymphomas, gastrointestinal, and gynecological cancers, as well as in those receiving total body irradiation for bone marrow transplantation—particularly those who may already be receiving statins. By preserving mitochondrial quality and limiting renal injury, ROSU could potentially reduce the risk of radiation-induced nephrotoxicity. Future clinical studies are warranted to evaluate its safety, efficacy, and optimal dosing in humans, which may inform strategies to protect renal function during radiation therapy.

Despite these findings, several limitations should be acknowledged. The current study focused primarily on the acute alterations in mitophagy prior to radiation exposure; however, it did not assess the long-term consequences that may emerge weeks or months after irradiation. Future investigations with extended follow-up periods are needed to assess fibrosis, chronic renal dysfunction, and potential recovery, which would provide more comprehensive and translational insights. Moreover, future studies would include measurements of mitochondrial membrane potential, ROS levels, and respiratory function to more accurately verify alterations in mitochondrial health.

## Conclusion

**Fig. 8:**
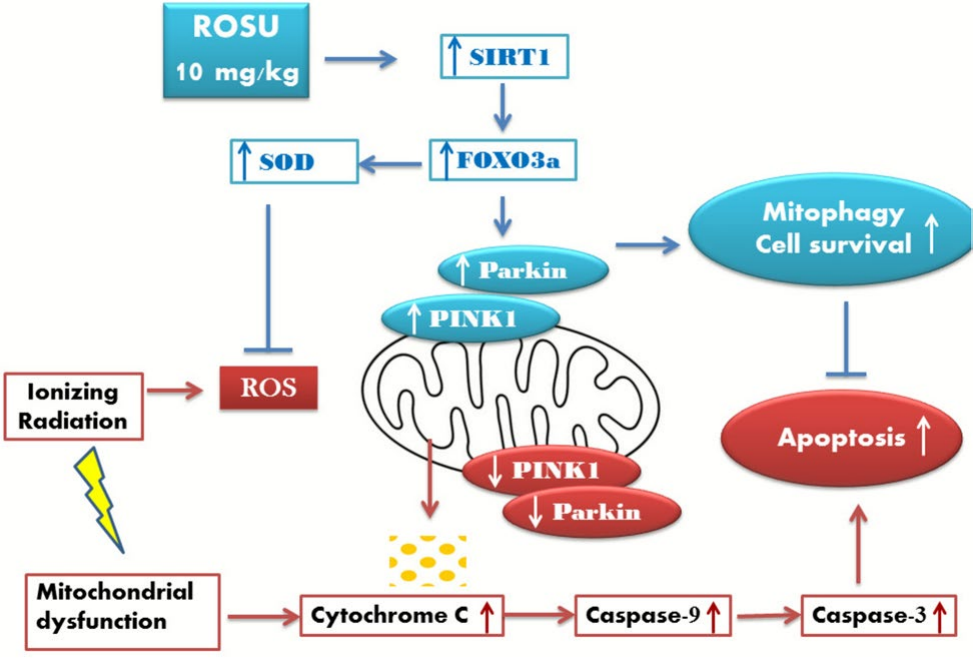
Schematic diagram showing the proposed mechanism of rosuvastatin (ROSU, 10 mg/kg) in mitigating radiation-induced nephropathy, highlighting its novel role in shifting cell fate from apoptosis toward mitophagy through activation of the SIRT1/FOXO3a signaling pathway. FOXO3a, forkhead box O3a; Parkin, Parkinson protein 2 E3 ubiquitin protein ligase; PINK1, PTEN-induced putative kinase 1; ROS, reactive oxygen species; SIRT1, sirtuin 1; SOD, superoxide dismutase

To sum up, the current study shed light on the nephroprotective impact of ROSU against acute and fractionated radiation exposure and demonstrated its mechanistic role in the activation of mitophagy machinery. Administration of ROSU activated the SIRT1/FOXO3a pathway which subsequently upregulated the anti-oxidant enzymes and mitigated the oxidative stress induced by radiation exposure. This led to minimizing mitochondrial ROS accumulation, thereby shifting the mitochondrial cellular response to the survival cascade by the activation of mitophagy proteins PINK1/Parkin and consequently, hindering the intrinsic apoptotic cascade that was triggered by radiation exposure. A schematic representation was carried out to show the proposed mechanism of ROSU in mitigating radiation-induced nephropathy, demonstrating the interplay between mitophagy and apoptosis (Fig. 8).

## Data Availability

The data that support the findings of this study are available from the corresponding author upon request.

## Abbreviations

AMPK: AMP-activated protein kinase
ATG5: Autophagy-related gene 5
BUN: Blood urea nitrogen
ELISA: Enzyme-linked immunosorbent assay
FOXO3a: Forkhead box O3a
GAPDH: Glyceraldehyde 3-phosphate dehydrogenase
GPx: Glutathione peroxidase
GSH: Reduced glutathione
Gy: Gray
IHC: Immunohistochemical
MDA: Malondialdehyde
NF-κB: Nuclear factor kappa B
PINK1: PTEN-induced kinase 1
Parkin: Parkinson protein 2 E3 ubiquitin protein ligase
ROS: Reactive oxygen species
ROSU: Rosuvastatin
SIRT1: Sirtuin1
SOD: Superoxide dismutase
